# The role of *Fusobacterium nucleatum* in the tumour microenvironment and carcinogenesis of oral and colonic malignancies

**DOI:** 10.1093/femsmc/xtag002

**Published:** 2026-01-10

**Authors:** Elisabetha Larionova, Gary P Moran

**Affiliations:** Smurfit Institute of Genetics, School of Genetics and Microbiology, Trinity College Dublin, Dublin, D02 VF25, Ireland; Dublin Dental Hospital and School of Dental Science, Trinity College Dublin, Dublin, D02 F859, Ireland

**Keywords:** *fusobacterium nucleatum*, microbiota, tumour microenvironment, colorectal cancer, oral cancer, dysplasia

## Abstract

The intra-tumoural microbiome is an increasing area of research with potential benefits in cancer diagnostics and treatment development. Numerous studies have implicated *Fusobacterium nucleatum*, a member of the oral microbiota, in the development, immune evasion, and dissemination of oral and colorectal tumours. Although *F. nucleatum* is yet to be classified as a cause or consequence of cancer, reports indicate the microorganism’s involvement in DNA damage, pathologic glucose uptake, and cellular proliferation. This accumulation of genetic instability is consistent with the multistep nature of malignant neoplasm progression. Virulence factors of *F. nucleatum* were shown to maintain an unresolved inflammatory state and impair the normal function of immune cells. The accompanying pro-inflammatory conditions facilitate vasculature remodelling, expediting tumour expansion, through a range of mechanisms. Pro-metastatic epithelial-to-mesenchymal transition and changes in gene expression have been observed in cancer cells upon *F. nucleatum* infection, suggesting an association with poorer prognosis. As a frequently encountered microorganism in the oral and colorectal intra-tumoural microbiome, *F. nucleatum* represents an intriguing, yet cautious research prospect with opportunities for novel prevention and therapeutic strategies. The objective of this work is to review the relevant evidence, taking into account the complexity of the tumour microenvironment.

## Teaser

Review and synthesis of the role in malignancies, genomic analysis, and therapeutic potential of a Gram-negative oral pathogen, *Fusobacterium nucleatum*.

## Introduction

**Figure 1:**
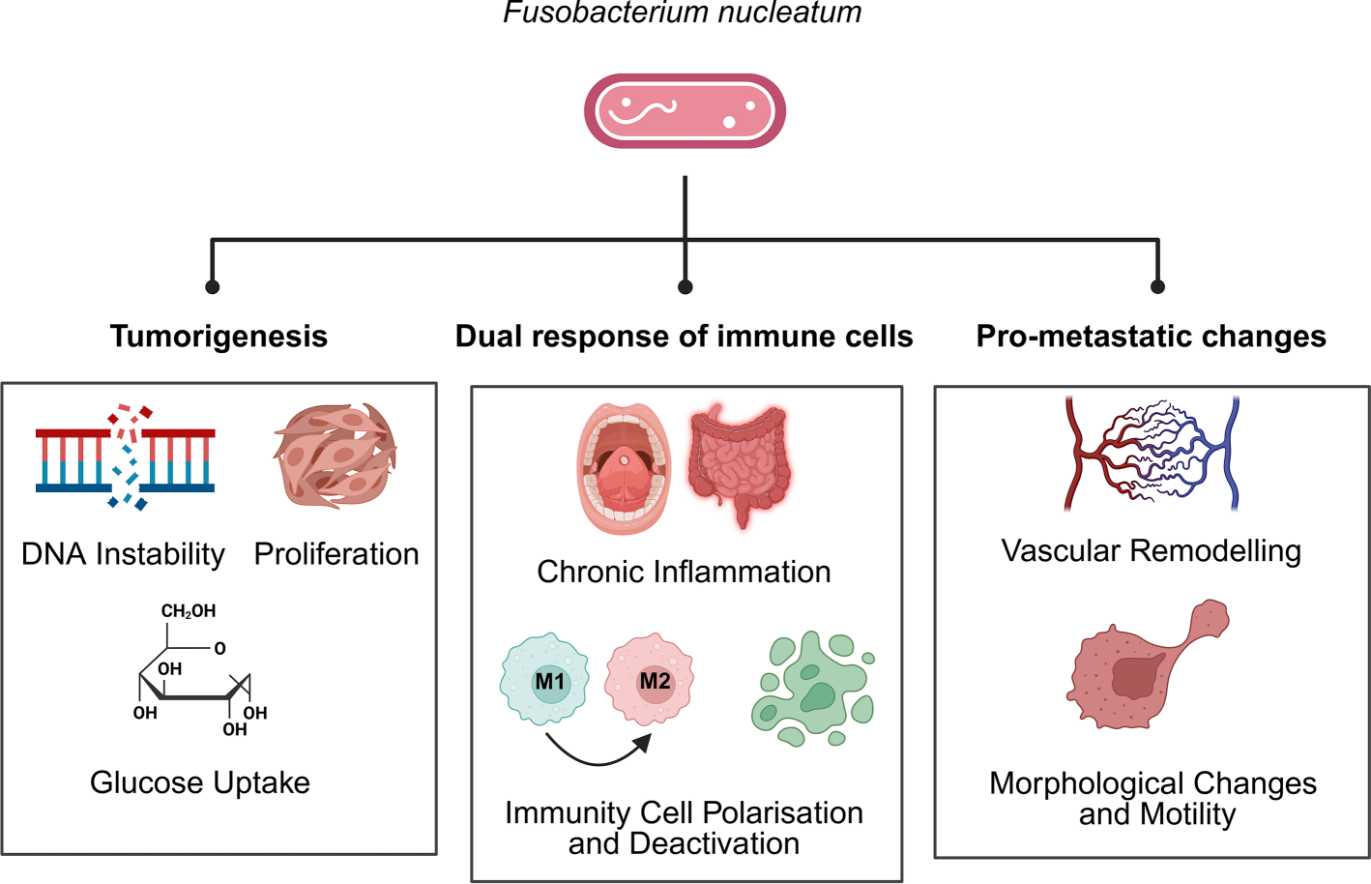
A graphical summary of the processes and events impacted by *Fn* within the TME of OCC and CRC. These include DNA instability, glucose uptake, and proliferation as parts of tumourigenesis; chronic inflammation, immune cell polarization, and deactivation as parts of pro-inflammatory and anti-tumoural response; vascular remodelling, morphological changes, and motility as parts of pro-metastatic changes.

Similar to other malignancies, oral cavity (OCC) and colorectal (CRC) cancers are a consequence of a multistep process involving various genetic and epigenetic alterations. The underlying risk factors of these alterations vary in OCC and CRC, but tobacco consumption, low antioxidant intake, and heritability are common factors in these conditions (Johnson et al. 2013, Chamoli et al. 2021). The gradual accumulation of molecular defects, such as chromosomal abnormalities, loss of heterozygosity for tumour suppressor genes, and histone modifications, is one of the major alterations that result in these conditions (Schmitt and Greten 2021, Georgaki et al. 2021). Recent genomic analyses further focus on the impact of the intra-tumoural microbiota—an intrinsic component of the tumour microenvironment (TME) (Zepeda-Rivera et al. 2024). *Fusobacterium nucleatum* (*Fn*) has been identified as one of the most dominant genera in the TME of both OCC and CRC, enhancing cancer progression and heterogeneity (Niño et al. 2022). *Fusobacterium nucleatum* is an opportunistic Gram-negative anaerobic bacillus mainly found in the human mouth and several parts of the gastrointestinal tract. Unlike other cancer-associated microorganisms, *Fn* encode distinct virulence factors that are suggested to promote genetic instability and hallmarks of cancer (Brennan and Garrett 2019). This review focuses on the roles of *Fn* as a member of the intra-tumoural microbiota in carcinogenesis (Fig. 1). Specifically, aspects that facilitate cancer cell development, survival, and dissemination will be discussed.

### CRC and OCC—prevalence and pathogenesis

In 2024, CRC accounted for 7%–8% of estimated new cancer cases in women and men, respectively (Siegel et al. 2024), making this condition one of the leading cancer diagnoses in developed countries like the United States. Among the factors mentioned previously, smoking, red meat intake, higher body mass index, and heredity significantly correlate with CRC (Johnson et al. 2013). Most CRC cases arise from a polyp or a neoplastic precursor lesion that contains genetically unstable stem cells or cells alike, with most progressing into cancer asymptomatically if not investigated (Dekker et al. 2019). Associated conditions, such as inflammatory bowel disease (IBD), frequently contribute to the CRC symptom complex (Johnson et al. 2013), which could cause atypical bowel habits, abdominal pain, rectal bleeding, weight loss, and anaemia (Dekker et al. 2019). Analysis of the microbial composition of human CRC with metagenomics revealed *Fn* to be the most abundant bacterial species, contributing to over 20% of total bacterial sequences found in the TME. Moreover, the bacterium has been linked to the ‘alpha-bug concept’, referring to an organism which, beyond harbouring pro-carcinogenic factors, can remodel the microbiome entirely to favour disease progression (Kostic et al. 2012). This and compelling findings on the association between *Fn*-specific virulence factors and CRC development (Guevarra et al. 2018, Rubinstein et al. 2019, Queen et al. 2025) highlight the significance and potential of this microbe as a research subject.

OCC is less common than CRC, constituting 3% of all new cancer cases in the United States, although the incidence of several of its subtypes is experiencing a consistent rise (e.g. human papillomavirus (HPV)-associated OCC, by 2.3% per year) (Siegel et al. 2024). The spectrum of risk factors of OCC is relatively narrow, including tobacco consumption, alcohol, and HPV infections. Still, the heredity of genetic alterations has the strongest correlation (Chamoli et al. 2021). Oral neoplasms are destructive masses, with most originating from tissue changes known as proliferative verrucous leukoplakia (i.e. white patches) and erythroplakia (i.e. red lesions) (Watanabe et al. 2015). Most OCC cases are diagnosed at a late stage when cancer spreads to the lymphoid tissue of the neck. The reason for this is the main clinical feature of OCC—pain, which arises once tissue changes reach significant proportions (Chamoli et al. 2021). In parallel with CRC, comparative evidence for OCC suggests *Fn* to be the most prevalent species in the TME, contributing to 47% of bacterial sequences (Bronzato et al. 2020). This increase in the organism’s relative abundance in OCC compared to CRC could be attributed to the fact that *Fn* is a permanent resident of oral microbiota, as outlined earlier.

## The role of *Fn* in tumourigenesis & tumour progression

Although there is debate about whether *Fn* is a pro-carcinogenic bacterium or a consequence of cancer, the literature repeatedly demonstrates its presence and sometimes dominance in the TME of major human cancer types (Zepeda-Rivera et al. 2024). For instance, as a component of TME, *Fn* has been reported to affect the biology of distinct cellular mechanisms, facilitating tumourigenesis. There are three key aspects linking *Fn* with this transformation, which have been interchangeably observed in the OCC and CRC cases.

### Accumulative DNA damage

**Figure 2:**
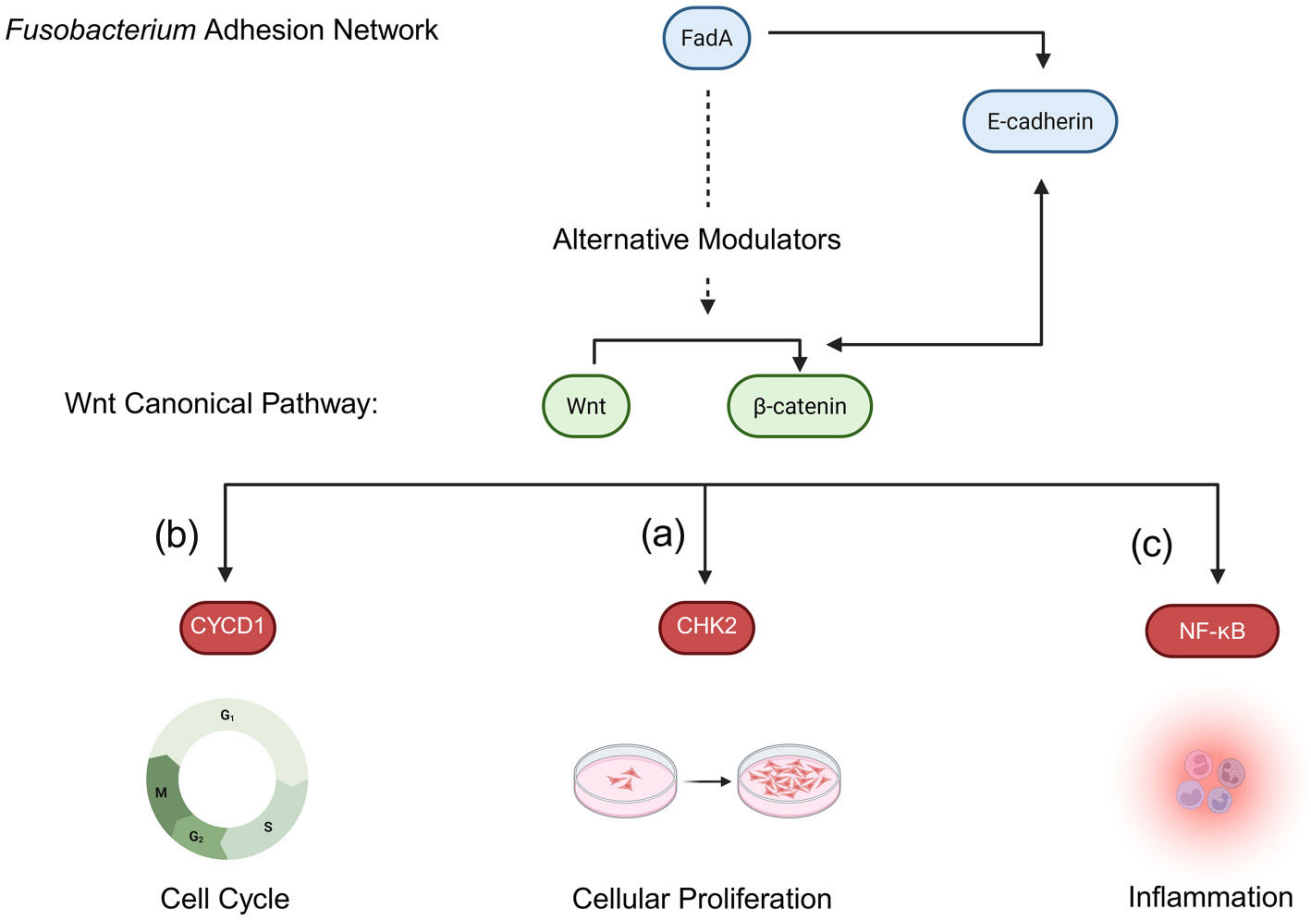
A Wnt signalling pathway summary depicting the link between Wnt-*β*-catenin signalling and FadA, produced by *Fn*. A dotted line represents a connection that was not explored in this review but can be seen throughout the literature. (a) The cell cycle-altering agent checkpoint kinase 2 (CHK2) is affected by the canonical Wnt-*β*-catenin signalling. (b) The cellular proliferation inducer cyclin D (CYCD1) is one of the direct targets of *β*-catenin within the Wnt pathway. (c) The pro-inflammatory gene family, NF-κB, also represents one of FadA's downstream targets within the depicted cascade.

The ability of *Fn* to cause different types of DNA damage, including double-strand breaks (DSBs), supports the bacteria’s role in tumourigenesis, as the accumulation of DNA damage is closely associated with the hallmarks of cancer. Since the 1990s, numerous studies have demonstrated the importance of DNA fragility, its impaired repair signalling, altered cell cycle checkpoints and replication fidelity in cancer. DSBs are considered the most severe form of DNA damage if not repaired by the cell (Huang and Zhou 2021). For instance, DSBs can be repaired by non-homologous end-joining, initiated by a Ku70 and Ku80 heterodimer structure in synergy with the P53 tumour suppressor protein. After confirming the presence of DSBs with the $\gamma $H2AX molecular marker, researchers observed the inability of Ku70 to repair the *Fn*-induced DNA damage in oral squamous cell carcinoma (OSCC). The lack of normal Ku operation results in P53 downregulation, enriching the mechanisms of OSCC growth and development (Geng et al. 2020). Other modes of damage are possible: specific CRC subtypes exhibit microsatellite instability-high (MSI-H), also known as *CpG* island hypermethylation phenotype. MSI-H tumours possibly arise from the defects in or complete loss of DNA mismatch repair proteins, such as MutL homologue 1 (MLH1), MutS homologue 2 (MSH2), and epithelial cell adhesion molecule (EPCAM), resulting in microsatellite DNA or repetitive sequences with errors (Chang et al. 2020). Correlation Analysis of MSI-H in human CRC tumours and *Fn* abundance performed by Okita et al. (2020) observed that heavy or moderate loads of *Fn* DNA were associated with this type of genetic alteration, although no specific mechanism is currently known. Moreover, activation of the *Fusobacterium* adhesin (FadA) produced uniquely by *Fn* and its binding to epithelial (E)-Cadherin within the Wnt-$\beta $-catenin pathway also plays a role in DNA damage by upregulating checkpoint kinase 2 (CHK2) (Fig. 2a). This kinase has been previously implicated in various cancers as a cell cycle agent responsible for its delay and CHK2-mediated DNA damage. Blotting evidence demonstrated the inability of FadA^−/−^  *Fn* to affect CHK2, reducing the bacterial biomass and tumour load in the murine colon (Guo et al. 2020). Unrepaired DNA damage and genetic instability induced by *Fn* predispose cells to mutagenesis and transformation. Consequently, *Fn*-associated DNA damage has been documented in both oral and colorectal malignancies, characterized by the occurrence of DSBs and MSI-H. The bacterial adhesin FadA promotes this instability even further, thus enhancing the mutagenic effects of *Fn*.

### Elevated glucose uptake

The presence of *Fn* correlates with glycolysis—the major nutritional source for malignant tumours and a recognized hallmark of cancer. Specifically, this process is fundamental for tumoural cell survival, growth, and metastasis. To promote colonization among the CRC cells, *Fn* mediates the expression of angiopoietin-like 4 (ANGPTL4), which is associated with elevated glucose uptake and hypoxia. Upon discovery of a positive correlation between the levels of ANGPTL4 and the abundance of the *Fusobacterium* genus, research demonstrated that this glycoprotein’s repression via knockdown led to a decreased *Fn* biomass (Zheng et al. 2021). High ANGPTL4 expression has been previously noted in oral malignancies with poorer prognosis (Hu et al. 2011), but recent studies have focused on a different set of glycolysis-related genes that have screening potential (e.g. *DEPDC1*) (Huang et al. 2023). Among other mechanisms, *Fn* activates the transcription of long non-coding RNA (lncR)-ENO1-IT1 by increasing the binding efficiency of the SP1 transcription factor (TF) to its associated promoter region. The ENO1-IT1 transcripts specify the histone modification pattern on the *ENO1* gene essential for glycolysis, as illustrated by the lack of lactate production in the case of this gene’s knockdown (Hong et al. 2020). Unlike for CRC cells, this specific mechanism is yet to be investigated in OCC. Still, reports contain another gene essential for glycolysis—*GLUT1*, known as a determining switch for tumoural metabolism. Here, *GLUT1* is afflicted by the *Fn*-activated *N*-acetylgalactosamine (GalNAc)-autophagy-TBC1 domain family member 5 (TBC1D5) signalling. Findings by Sun et al. (2023) suggest that *Fn* requires GalNAc for adhesion in the OSCC cells, with the outcome of this selective binding being autophagy, as illustrated by autophagy markers (e.g. *BECLIN-1*). The connection with *GLUT1* arises from autophagy or its activation with synthetic compounds (e.g. 3-methyladenine), which downregulates the TBC1D5 signal modulator required for regulated glycolysis. TBC1D5 degradation in *Fn* infection increases GLUT1 levels and localizes it on the cell surface, as validated by the elevated deposition of extracellular lactate (Sun et al. 2023). Even though many of the *Fn*-stimulated glycolytic pathways are described today, capturing the entire molecular network remains a challenge. While *Fn*-directed upregulation of glycolysis has been reported in both cancers, emerging evidence suggests that the specific mechanisms might not be uniformly observed across OCC (e.g. upregulation of ANGPTL4 and GLUT1) and CRC (e.g. lncR-ENO1-IT1 transcription). Whether these differences reflect gaps in current research or represent genuine biological deviations in metabolic regulation remains to be determined.

### Abnormal cellular proliferation

Lastly, *Fn* binding and invasion upregulate the oncogene expression through several pathways. Rubinstein et al. found that the binding of E-Cadherin by the previously described FadA adhesin may also upregulate Annexin A1–a recently recognized modulator of the Wnt-$\beta $-catenin signalling (Rubinstein et al. 2019). In this case, transfection of colon carcinoma cells with *ANXA1*, encoding Annexin A1, increased the expression of cyclin D1 (CYCD1) (Fig. 2b)—a determinant of uncontrolled cellular proliferation (Montalto and De Amicis 2020). Similar findings were observed in mice orally infected with *Fn*. Their tongue tumours were 2.5-fold larger and more invasive, statistically correlating with elevated CYCD1 (Gallimidi et al. 2015). Further evidence is provided by the studies focusing on microRNA-21 (miR-21) as a downstream target of *Fn* in the colon, upregulated at the transcription level in response to the toll-like receptor (TLR)-4 signalling to MYD88. Upon *Fn* infection, TLR-4 and MYD88, participants of the canonical nuclear factor (NF)-$\kappa $B pathway branch, cause a consequent binding to the promoter of cancer-enhancing miR-21, as illustrated by chromatin immunoprecipitation (Yang et al. 2017). The interaction between TLR-2 and oral epithelial cells also stimulates growth, as inhibition of the receptor’s signalling via neutralising antibodies abolished the effect of *Fn* on OSCC proliferation. Several of the agents underlying this change were CYCD1, but also interleukins (ILs) and tumor necrosis factor (TNF)-$\alpha $ due to the immune cell infiltration (Gallimidi et al. 2015), discussed later. The examples listed here illustrate only a small portion of the tumourigenic pathways tied to *Fn* and cellular response. The ability of *Fn* to influence cellular proliferation through key regulators of the cell cycle (e.g. CYCD1) is evident in both oral and coloreactal tissues. As observed with its impact on glycolysis, pro-tumourigenic outcome is maintained, although the specific receptors and signalling involved may differ.

## The role of *Fn* in tumour-promoting inflammation & anti-cancer immunity

As illustrated by the disorders predisposing to CRC and OCC, such as IBD and chronic periodontitis (Wu et al. 2019), the inflammatory microenvironment favours tumour progression and survival. On the one hand, unresolved or chronic inflammation promotes malignant structures by facilitating the accumulation of tissue and DNA damage. On the other hand, preventing the normal function of active immunity cells is equally valuable for the tumour to maintain its immunosuppressive state (Wu et al. 2019). Stimulation of pro-inflammatory factors and manipulation of both innate and adaptive immunity cells by *Fn* has been repeatedly demonstrated in OCC and CRC.

### Chronic inflammation

As numerous *Fn*-specific inflammatory response signatures exist, this work primarily concentrates on pro-inflammatory mechanisms observed in IBD and chronic periodontitis. The importance of these conditions lies in their definition as long-term risk factors for cancer, given their chronic inflammatory nature. Moreover, *Fn* isolates retrieved from inflamed tissues have been previously shown to invade healthy tissues faster (Strauss et al. 2011), amplifying their role in creating an inflammatory microenvironment for the tumour. Table 1 summarizes the contribution of *Fn*-associated virulence factors to chronic inflammation.

**Table 1:** Virulence factors of *Fusobacterium nucleatum* and the respective mechanisms for their impact on inflammation.

Virulence factor	Respective mechanism(s)	References
*Fusobacterium* adhesin/invasin A (FadA)	FadA is required for the stimulation of E-Cadherin, which is correlated with the expression of the NF-$\kappa $B and IL-6/-8/-18 cytokines. Moreover, an increase in *FadA* expression was consistent with the expression of the *Wnt7b—*Wnt gene and *NF*$\kappa $*B2* (Fig. 2c).	Rubinstein et al. 2013, Gallimidi et al. 2015 Rubinstein et al. 2013, Gallimidi et al. 2015
Fibroblast activation protein 2 (Fap2)*	Fap2 is a galactose-inhibitable adhesin which binds the overexpressed GalNAc on the CRC cell surfaces. Fap2-dependent *Fn* invasion resulted in high secretion of IL-8 and chemokine C-X-C motif ligand 1 (CXCL1), associated with tumour growth, metastasis and poorer prognosis.	Casasanta et al. 2020
Arginine- inhibitable adhesin (RadD)*	RadD interacts with the CD147 receptor, overexpressed on the CRC cell surfaces. Preliminary studies noted that this binding can activate the NF-$\kappa $B and phosphoinositide 3‐kinase—both implicated in uncontrolled inflammation.	Zhang et al. 2024
Endotoxic lipopolysaccharide (LPS)	LPS, found in the outer membrane of *Fn*, can be recognized by TLR-4, which stimulates the NF-$\kappa $B pathway through the previously mentioned TLR-4/MYD88 cascade. Involved pro-inflammatory cytokines include but are not limited to IL-6 and TNF-$\alpha $.	Gallimidi et al. 2015
Outer membrane vesicles (OMVs)	Being released by *Fn* in the extracellular space, OMVs utilize ligand-receptor interactions to elicit signalling. Their addition to the epithelial cells stimulates the secretion of IL-8 and TNF through the effectors, including NF-$\kappa $B. Treatment of oral tissue M1 macrophages with OMVs demonstrated elevated expression levels of IL-1$\beta $/-6 and TNF-$\alpha $.	Engevik et al. 2021 Chen et al. 2022

Note: Compilation of the *Fn*-associated proteins or released agents that have been identified to substantially impact inflammation levels within the TME. A ‘*’ signifies that the respective mechanism is restricted to CRC, given the varying reporting in OCC.

IBD is a term used to describe conditions, such as Crohn’s disease (CD) and ulcerative colitis (UC), that emphasize the importance of host-microbe interaction due to their underlying causes. For instance, the previously described interaction of *Fn* with E-Cadherin has been proposed as a possible mechanism for enhancing the permeability and fragility of the intestinal barriers in CD (i.e. tight and adherent cell-cell junctions). Once the epithelial cells are infected, the bacteria impact the CD4^+^ T cell differentiation, which results in the overproduction of pro-inflammatory cytokines: interferon (IFN)-$\gamma $, TNF-$\alpha $, IL-1$\beta $/-6/-17 (Liu et al. 2020). Similarly, the properties of *Fn* as a potent activator of the NF-$\kappa $B pathways have been observed in intestinal epithelial cells and similar models. The release of extracellular heptose-related metabolites by CRC-associated *Fn* through the Alpha Kinase 1/TIFA pathway activated the NF-$\kappa $B signalling, shown by the combination of luciferase reporter assay and gene knockdown in HT29 cells (Martin-Gallausiaux et al. 2024). These events are pivotal in inflammation, as this cascade regulates cytokine production (Liu et al. 2017). NF-$\kappa $B activation is also observed in UC, where *Fn* is defined as one of the most abundant pro-inflammatory species found in the patients’ epithelial and submucosal tissues at ∼42% higher than in health (Chen et al. 2020). The multi-step activation initiates with the targeted caspase activation and recruitment domain 3 (CARD3)—a serine/threonine/tyrosine kinase, by *Fn*. Although Chen et al. (2020) did not describe a specific mechanism, the researchers reported a positive correlation between the *CARD3* mRNA expression and the abundance of *Fn*. A similar trend has been observed for this cascade’s members NOD2 and TLR4, which recognize CARD3 and their downstream target—IL-17F. These steps result in the NF-$\kappa $B stimulation observed in both CD and UC, supported by their symptomatic and molecular similarities.

Advanced and irreversible forms of periodontitis, characterized by soft tissue degradation and tooth loss due to chronic inflammation of the gingival epithelium, frequently share the presence of *Fn* in the diseased periodontal pocket. Inflammatory outcomes are illustrated by the immune response of gingival epithelial cells that serve as a mucosal barrier to infection. Specifically, the mRNA expression and production of pro-inflammatory cytokines, such as IL-1$\beta $/-6/-17 and IFN-$\gamma $, at high levels followed *Fn* infection of murine gingiva and prompted immune cell infiltration and bone resorption, as seen from tissue histopathology. Further upregulation of TNF-$\alpha $ and HMGB1, a damage-associated molecular pattern molecule, was consistent with the activation of TLR-2/-4 (Johnson et al. 2018). Gingival fibroblasts, the predominant cell type of periodontal tissue, represent another cell type affected by *Fn*-induced inflammation. Similar to the *Fn* infection in the gingiva, activation of *TLR-2/-4* genes promotes a statistically significant increase in the release levels of IL-6/-8 in fibroblasts within the first 48 h. Moreover, treated fibroblasts displayed a dose- and time-dependent increase in intracellular radical oxygen species (ROS) production, as determined by flow cytometry (Kang et al. 2019). ROS and NF-$\kappa $B TFs have common but complex interactions, where ROS are reported to both activate and repress this kind of signalling. Nonetheless, many NF-$\kappa $B enzymatic targets represent ROS-producing enzymes, such as NADPH oxidase 2 and cyclooxygenase-2, which are well-known for their modulation of immune responses (Morgan and Liu 2010). A different type of resident periodontal cells—gingival keratinocytes have also been found to produce a strong antimicrobial and inflammatory response upon interaction with *Fn* structures. Following the incubation of the Ca9-22 keratinocytes with the *Fn* cell wall extracts, researchers observed a two-fold increase in IL-1α and IL-6 mRNA, along with a multi-fold increase in IL-8 and MMP-9 mRNA, likely mediated through TLR-2 (Peyret-Lacombe et al. 2009). The release of pro-inflammatory cytokines induced by *Fn* has been documented in both colorectal (e.g. epithelial and submucosal) and oral (e.g. gingival) tissues. This inflammatory activity, often associated with chronic conditions that precede malignant neoplasms, plays a central role in mediated structural damage and a dysbalanced immune response.

### Macrophages

Macrophages, abundant in almost every body tissue, are involved in numerous biological processes due to their ability to differentiate and adapt. In health, tissue-resident macrophages work towards maintaining homeostasis and tissue repair. A pathology results in monocyte-derived macrophages that are phenotypically distinct and meant for host defence. Modulation of the inflammatory microenvironment within the tumour by *Fn* also manifests in the amplification of bone marrow-derived cells like tumour-associated macrophages (TAMs). Specifically, the bacteria affect macrophage heterogeneity, promoting its differentiation from a pro-inflammatory M1-phenotype to a TAM M2 phenotype, as detected by the immunofluorescent characterization. In addition to being the predominant infiltrating immune cell population in human *Fn*-containing CRC, CD68^+^ macrophages challenged with *Fn in vitro* and *in vivo* predominantly displayed the TAM phenotype. The M2-polarization is induced in a TLR-4-dependent manner, possibly involving IL-6, p-STAT3, and c-MYC TFs due to their increased co-expression and colocalization with TAMs (Chen et al. 2018). Similarly, TAMs are utilized by oral malignancies to maintain immunological self-tolerance and promote tissue reconstruction. Nie et al. (2024) argued that M2-polarization could be prompted by the secretion of C-X-C motif ligand 2 (CXCL2), observed in *Fn*-infected OSCC cells. Following *Fn* stimulation, this chemokine exhibited the highest expression level among the tested chemokine families, while its silencing sensibly attenuated macrophage recruitment and polarization (Nie et al. 2024).

The impact of *Fn* on macrophages extends further, as its main metabolite, butyric acid (BA), has been implicated in macrophage deactivation and impaired maturation. BA is a naturally occurring compound produced by bacterial anaerobic fermentation, typical of the intestinal microflora. Isolated and *in vivo* intestinal macrophages treated with n-butyrate demonstrated a reduced gene expression of pro-inflammatory mediators, such as *NOS2, IL-6*, and *IL-12a*/*12b*. A theoretical cause of n-butyrate action concerns its role as an immune system sensor in health that prevents macrophages from attacking beneficial, n-butyrate-producing bacteria (Chang et al. 2014). Studies of the oral cavity where BA is of pathologic occurrence suggest that periodontopathic bacteria compromize the innate immune system by affecting the macrophage precursor cells—monocytes. Past studies report a low dose of n-butyrate of 0.5 mM inhibiting the cytokine- or lipopolysaccharide (LPS)-driven differentiation of isolated human monocytes. Since the free fatty acid G-protein coupled receptor 43 is highly expressed in immune cells and monocytes in particular, this receptor might be the major contributor to BA toxicity (Abe 2012). Further effects of *Fn* on macrophages, including cell invasion and the expression of indoleamine 2,3-dioxygenase on the cell surface to escape immunosurveillance, have been observed (Xue et al. 2018). These immunosuppressive strategies to ensure a tumour-favouring environment are not limited to macrophages and similarly encompass numerous cells of innate immunity. Across CRC and OCC, *Fn* exerts comparable deactivating and impairing effects on macrophage function. This convergence highlights macrophages as one of the key cellular targets of the microorganism and underscores the ‘alpha-bug concept’ introduced earlier.

### Neutrophils, natural killer, and dendritic cells

Innate immunity comprises several cell lineages, with neutrophils, natural killer (NK) and dendritic (DCs) cells contributing towards the changes observed throughout cancer progression. Neutrophils are granulocytes that are essential for host defence due to their abilities to consume foreign media, release lytic enzymes and produce pro-inflammatory factors. In periodontitis and CRC, *Fn*-infected and inflamed tissues were shown to contain significantly higher amounts of hyperactive neutrophils compared to the control. For instance, biofilm-grown *Fn* stimulated four–six-fold higher total ROS release by neutrophils, reported to occur faster than in controls (Muchova et al. 2024). The build-up of ROS-induced oxidative damage and lack of its timely repair due to neutrophil hyperactivity are toxic and can promote TME cell survival. *Fn* also induces neutrophil extracellular trap (NET) production, stimulated by persistent inflammation and cellular stress, as determined by the NET formation and purification analysis. This antimicrobial defence is abundant in OSCC and CRC tumours and confers the activation of TLR4-ROS, IL-17, and proliferative NOD1/2 gene expression (Garley 2023, Kong et al. 2023). Moreover, correlation analysis between the bacterial short-chain fatty acid concentration and the transforming growth factor (TGF)-$\beta $ suggested that *Fn*-generated butyrate promotes TGF-$\beta $ expression (Martin-Gallausiaux et al. 2018). TGF-$\beta $ is a key signalling component whose role in neutrophil polarization towards the pro-tumourigenic N2 phenotype is still emerging. Specifically, results in various models suggest that TGF-$\beta $ affects neutrophil cytotoxicity, endothelial adhesiveness, and release of the following pro-inflammatory substances. The consistent rise in metalloproteases (MMPs)-2/9, vascular endothelial growth factor (VEGF), and proliferative markers (e.g. Ki67) production by neutrophils results in enhanced migratory and angiogenic activities of tumour cells, which is indicative of poorer survival (Muchova et al. 2024, Kong et al. 2023).

The impact of *Fn* on mononuclear cells, including NK and DCs, diminishes anti-tumour immunity. In health, NK cells respond to infected structures by releasing perforin and granzyme granules required for assisted killing. To prevent chronic inflammation and autoimmune disorders, NK cells are tightly regulated by activating and inhibiting receptors on their surface, which respond to tumour proteins, self-molecules, and virulence factors (Koch et al. 2013). An exemplary co-inhibitory receptor is T cell immunoglobulin and immunoreceptor tyrosine-based inhibitory motif domain, or TIGIT. Zhang et al. (2018) observed that TIGIT expression in tumour-infiltrating NK cells leads to malignancy growth and substantially reduced occurrence of perforin, IFN-$\gamma $, and TNF, as observed from the combination of cytokine staining and flow cytometry. Importantly, the most abundant membrane agent of *Fn*, fibroblast activation protein 2 (Fap2), is known for binding TIGIT, allowing a non-targetable tumoural growth in CRC and most likely OCC, as fewer investigations assessed this protein’s potential in the oral cavity (Guevarra et al. 2018).

Similar to neutrophils, antigen-presenting DCs either dampen or promote lymphocyte invasion. DCs were shown to promote cytotoxic CD3/8^+^ lymphocyte suppression in the colon by elevating the immunosuppressive CD4^+^ and FOXP3^+^ T regulatory cell concentration post-*Fn* treatment (Kostic et al. 2013). Interestingly, immunohistochemistry studies of OSCC noted CD103^+^ DCs as markers of the opposite, favourable prognosis due to their presentation as stromal tissue-resident memory T cells (Xiao et al. 2019). Nonetheless, *Fn* samples correlate with another surveillance escape mechanism in OCC—upregulation of an inhibitory receptor ligand widely expressed on numerous cells, known as programmed death-ligand 1 (PD-L1) (Michikawa et al. 2022). In this scenario, PD-L1 can be used as a negative feedback measure by DCs to control CD8^+^ lymphocyte reactivation and protect DCs from cytotoxicity, dampening anti-tumour immunity and reducing the tumour-infiltrating cell populations. The mechanisms of *Fn* to deviate innate immunity are vast and consequently impact the efficiency of adaptive systems due to imminent crosstalk. While consistent findings have established neutrophils as cellular targets of *Fn* in both cancer types, comparable evidence in NK cells and DCs remains limited. Resolving the mentioned gaps and inconsistencies through targeted investigations could provide valuable insights for the development of the innate system’s therapeutic modulation in response to the pathogen.

### T lymphocytes

**Figure 3:**
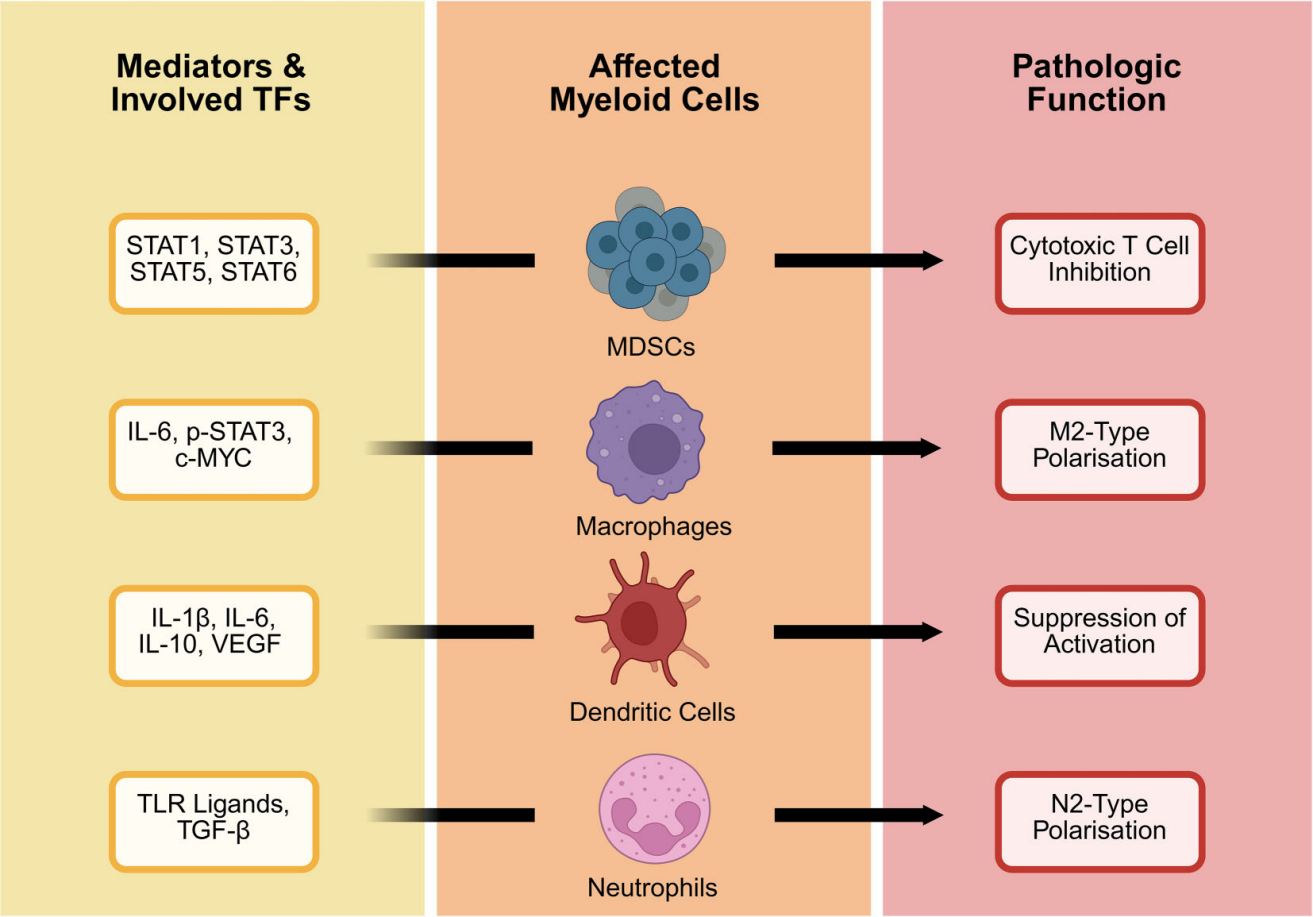
A summary of the myeloid cells (i.e. originating from a multipotent haematopoietic stem cell, such as macrophages, DCs, and granulocytes) affected by the TME, involved mediators, and the resulting pathologic functions. The effects of these changes in transcription and MDSC levels extend to the cells of adaptive immunity.

*Fn*-enhanced inhibition of cell killing by cytotoxic and effector T lymphocytes, as outlined earlier, is not a single-mechanism occurrence. Two more examples of such interference, driven by *Fn* interactions and promoting immune escapism, are represented by the general recruitment of myeloid-derived suppressor cells (MDSCs) and the CC chemokine ligand 20 (CCL20)-CC receptor 6 (CCR6) pathway. Differentiation of myeloid cells, as outlined previously, is diverted by the TME, generating immature and pathologic MDSCs (Fig. 3). Directed by abundant TFs, such as the signal transducer and activator of transcription (STAT) family members, MDSCs deprive T cells of amino acids essential for growth (e.g. L-cysteine, L-arginine), and induce T cell apoptosis via the galectin 9 (GAL9)-T cell immunoglobulin and mucin domain-containing protein 3 (TIM3) binding (Gabrilovich et al. 2012). OSCC and CRC models treated with *Fn* supported the connection between the enhanced MDSC recruitment and lowered density of CD3^+^ via a tissue array or CD8^+^ T cell proliferation via an activation assay, respectively (Mima et al. 2015, Zhong et al. 2019). Interactions between chemokines and their receptors, selectively expressed on T lymphocytes, further complicate the TME. For instance, Rutihinda et al. (2022) correlated chemokine CCL20 with the levels of intra-tumoural T regulatory and pro-inflammatory T helper 17 cells, selectively expressing CCR6, which suppressed the migration and activation of cytotoxic CD8^+^ lymphocytes. Although evidence suggests that *Fn* elevates the CCL20+ cell number, especially in tumour-favourable conditions (e.g. hypoxia), understanding of the CCL20-CCR6 axis throughout the entire progression of the OCC and CRC disorders is not complete (Mandal et al. 2021).

Lastly, the versatility of immunosuppressive methods exhibited by *Fn* to avoid immune recognition is supported by the lack of tumour-infiltrating lymphocytes (TILs) (e.g. CD3/8^+^ cytotoxic, CD4^+^ helper, CD45RO^+^ memory, and CD19^+^ B-cells) in OCC and CRC. The amount of *Fn* DNA in tumour tissue has a negative correlation with TIL density and gene expression, as illustrated by large cohort studies (Borowsky et al. 2021, Singh et al. 2023). This evidence also describes a statistically significant reduction in the proportion of antibody-secreting B-cells in cancer patients by two-fold compared to controls. However, this currently lacks specific *Fn*-directed mechanisms. Taken together, the available evidence suggests that most of the virulence factors produced by *Fn* and exported directly or via outer membrane vesicles (OMVs) contribute to chronic inflammation (Table 1) play roles in the cascades where the cells from both innate and adaptive immunity get polarized towards a tumour-promoting phenotype or deactivated, maintaining a balance between inflammation and immunosuppression. In contrast to the cells participating in the innate response, most components of the adaptive immunity, except B-cells, for which the lack of pre-clinical data becomes apparent, look to be influenced by similar means in both CRC and OCC. These unique signatures, amplified by the *Fn* infection, accompany two events underlying malignant neoplasm expansion and spread—angiogenesis and metastasis.

## The role of *Fn* in angiogenesis & metastasis

In the absence of appropriate immune surveillance due to the TME advancement by *Fn*, malignant neoplasms expand and spread to the nearby or metastatic sites. When analysing the primary-metastatic tumour pairs, *Fn*-positive primary tumours result in the presence of *Fn* nucleic acids in the lungs (75% occurrence rate) and bone in OCC; liver (64% occurrence rate) and lymph nodes in CRC (Bullman et al. 2017, Chen et al. 2024). From murine models to patient records, evidence suggests a correlation between high-level *Fn* abundance in faecal specimens, positive lymph node metastasis, and greater depth of invasion in CRC patients (Chen et al. 2022). Fewer epidemiological data are available in OCC, and current findings do not reach a consensus on whether oral metastasis can be predicted based on the presence of *Fn*. Survival analysis of HPV-negative tongue cancer patients revealed that the patients with a higher load of the bacterium had a poorer survival and an increased extracapsular spread, providing a rationale for larger-cohort associated studies similar to CRC (Desai et al. 2022). Angiogenesis, or the physiological process of new blood vessel formation from pre-existing vasculature present in both cancer types, represents a mechanistic link between chronic inflammation and the establishment of tumour metastases. The typical sequence of events involves stimulation of endothelial cells by factors released by the inflamed TME, degradation of the capillary basement membrane, and the migration of these cells, along with circulating cancer cells, to nearby sites. Even though the impact of *Fn* in CRC dissemination has been determined in theoretical models, corresponding biomarker validation or rapid-diagnostics assays remain unavailable, given the bottlenecks discussed subsequently.

### Vascular remodelling and tumour expansion

Essential for wound healing and tumour growth, angiogenesis is not inherently pathologic and involves endothelial cells, which are among the first to come in contact with *Fn* and its products. For example, *Fn* infection was found to increase the release of VEGF—the central angiogenesis-promoting agent secreted by the endothelial cells. Treatment of an OCC model with the bacteria illustrated two-fold changes in VEGF receptor expression, followed by VEGF release but not its mRNA expression (Mendes et al. 2016). This pattern is possibly due to *Fn* proteins only activating the autocrine function of endothelial cells, required for lumen-containing tube formation. *Fn* metabolite hydrogen sulfide (H_2_S) was shown to operate this mechanism at pathologic concentrations of 1 mM in CRC HCT116 cells (Wang et al. 2023). Pro-angiogenic factors are enhanced further under stressful conditions, such as hypoxia, due to the inefficient healing and inflammation within the TME. Mendes et al. (2016) treated an oral endothelium model with *Fn* to produce a sensible reduction in the cellular oxygen level of up to 15% and an upregulation of VEGF, IL-1$\alpha $, TNF-$\alpha $, and tube formation rates. Moreover, samples from oral potentially malignant disorders (i.e. leukoplakia and erythroplakia) demonstrated a significant abundance of *Fn* genera at 11.30%, supporting the link between the microorganism and malignant transformations in hypoxic conditions (Gurizzan et al. 2025). Although this mechanism is not currently described in CRC, reports confirm that *Fn* infection, CXCL1 and IL-8 cytokine secretion rates are higher in the hypoxia-conditioned model. Prolonged hypoxia, however, does not accelerate angiogenesis and is associated with a highly inflammatory environment, still promoting tumour expansion. Lastly, MMPs are fundamental for angiogenesis-dependent morphological changes and endothelial cell motility. ELISA and scratch wound assays of the *Fn*-infected OSCC H376 cells demonstrated a substantial increase in the MMP9 basal expression by three–five-fold and migration, respectively, also in the absence of E-Cadherin (Selvaraj et al. 2024). This finding’s significance is highlighted by the conventional role of E-Cadherin and $\beta $-catenin in the MMP9 expression activation to produce these changes, previously observed in *Fn*-positive CRC patients (Shi et al. 2022). Despite potential differences in MMP9 activation in OCC and CRC, MMP-9 remains a pathologic intravasation agent capable of matrix degradation and metastasis progression. While several mechanisms are shared (e.g. stimulation of VEGF release) and others remain only partially elucidated (e.g. *Fn*-induced hypoxia) in OCC and CRC, *Fn* appears to contribute to tissue remodelling, facilitating systemic spread and tumour advancement.

### Epithelial-to-mesenchymal transition

An essential feature of metastasis, enhanced by *Fn*, is the epithelial-to-mesenchymal transition (EMT), observed in healing, inflammation, and malignant neoplasms. Throughout the EMT, epithelial cells lose their cell-cell adhesion and adopt a mesenchymal phenotype for improved motility. Yet, distinct changes in gene expression of tumour cells, presented further, allow an intermediate EMT state, where the mesenchymal phenotype and epithelial features are retained (Shao et al. 2021). The induction of these changes is affected by the *Fn*-associated factors during growth and infection, including OMVs in OCC and exosomes in CRC. The injection of purified OMVs into the OSCC xenograft caused an increase in tumour cell invasion and migration by two–three-fold, along with changes in the expression of the EMT markers, such as E-Cadherin and N-Cadherin. For instance, western blotting revealed time- and OMV concentration-dependent downregulation of E-Cadherin and upregulation of N-Cadherin via the autophagy pathway (Chen et al. 2024), recapped below. A similar trend is observed for the endocytosis-generated vesicles—exosomes, whose contents reflect genetic instability and microbial components of a tumour. The infection of the HCT116 line with *Fn* facilitated cellular secretion of exosomes, indicated by their accumulation, which promoted a morphological change and an increase in cell migration by 3-fold. Exosomal miRNA expression profile identified miR-1246/92b-3p/27a-3p as pro-metastatic agents, which elevated the tumour nodule appearance within the liver via Wnt-$\beta $-catenin pathway activation and a subsequent decrease in E-cadherin (Guo et al. 2021). Activation of the autophagy pathway by *Fn*, significant for EMT and cytoskeletal remodelling, has been shown in both cancer types. In OSCC, the expression of autophagy-related genes (e.g. *ATG3, ATG7, BECLIN-1*) and autophagosome synthesis increased after co-culturing the cells with *Fn* OMVs, indicating the pathway’s activation (Chen et al. 2024). In CRC, the same results were observed and attributed to the pro-metastatic CARD3. Transfection with CARD3 siRNA protected the cells from *Fn*-induced autophagy, E-cadherin downregulation, and metastatic gene signatures (Chen et al. 2020). The mechanisms employed by *Fn* within the TME, favouring its intracellular survival, are frequently associated with cancer invasion and chemoresistance, highlighting the importance of the bacteria’s detection and clinical consideration. A comparative summary of *Fn*-driven mechanisms in oral and colorectal malignancies is presented in Table 2, outlining pathways that are shared, differ, or are yet to be defined, as established in this review.

**Table 2:** A comparative summary of the functional hallmarks linked to *Fn* that are shared, differ, or are currently undefined between oral and colorectal cancerous disorders cited in this study.

Functional hallmark	Shared	Different	Undefined	References
Tumourigenesis and tumour progression	Induction of DSBs and MSI-H; increased expression of CYCD1 via *ANXA1* and TLRs.	Set of genes known to upregulate glycolysis—in OCC: *ANGPTL4, DEPDC1, GLUT1* and in CRC: *ENO1*.	None.	Geng et al. 2020 Okita et al. 2020 Rubinstein et al. 2019 Gallimidi et al. 2015 Zhang et al. 2018 Hu et al. 2011 Huang et al. 2023 Sun et al. 2023 Hong et al. 2020
Tumour- promoting inflammation and anti-cancer immunity	Polarization towards TAMs and N2 neutrophils; NET formation; MDSC recruitment; reduction in TILs.	Impact of DCs—in OCC: promoting lymphocyte activity and in CRC: dampening lymphocyte activity.	Repression of TIGIT in NK cells in OCC.	Chen et al. 2018 Nie et al. 2024 Garley 2023 Kong et al. 2023 Muchova et al. 2024 Xiao et al. 2019 Kostic et al. 2013 Zhong et al. 2019 Mima et al. 2015 Singh et al. 2023 Borowky et al. 2021 Guevarra et al. 2018
Angiogenesis and metastasis	Increased release of VEGF; activation of the autophagy pathway.	Activation of MMP9–in OCC: possible in the absence of E-cadherin. EMT-promoting vesicles—in OCC: OMVs and in CRC: exosomes.	Reduction in the cellular oxygen level in CRC.	Mendes et al. 2016 Wang et al. 2023 Chen et al. 2024 Chen et al. 2020 Selvaraj et al. 2024 Shi et al. 2022 Chen et al. 2024 Guo et al. 2021 Gurizzan et al. 2025

Note: The table only contains findings of the processes presented in this review.

## Research considerations & applications

Advances in next-generation sequencing led to the characterization of the TME microbiome and were followed by functional studies across cancer types, from early tissue transformation to metastatic dissemination, as highlighted in this review. Therapeutic and prognostic applications of this research are yet to be advanced due to a persisting gap between preclinical findings and the clinical implementation, complicated by factors such as strain-level and inter-species variation.

For instance, the development of common anti-*Fn* strategies against oral and colonic tumours can be complex, considering that *Fn* is a species complex consisting of four heterogeneous subspecies that are believed to differ in TME enrichment (Sivertsen et al. 2025). Out of the historically recognized subspecies—*F. nucleatum* (*Fnn*), *F. animalis* (*Fna*), *F. polymorphum* (*Fnp*), and *F. vincentii* (*Fni*), *F. nucleatum subsp. polymorphum* has been shown to exhibit greater prevalence in oral malignancies (Crowley et al. 2024). In contrast, in CRC, the enrichment of *Fna* was repeatedly observed in malignant neoplasms (Zapeda-Reivera et al. 2024, Queen et al. 2025). Zapeda-Rivera and colleagues (2024) et al. (2024) went further to propose distinct genetic clades of *Fna* associated with the oral cavity (*C1*) and CRC (*C2*). However, this logical distinction highlights the complexity of *Fn* phylogenetics, as the *C1* designation of *Fna* has since been disputed by Silversten et al. (2025), who have proposed reclassification of *Fn C1* as the rare species *F. watanabe*. The utility of strain designations is further complicated when the distribution of proposed virulence genes is examined. For instance, not every strain of *Fn* possesses the proposed virulence factors in Table 1, such as Fap2 or RadD, and even when present, these genes often exhibit evidence of extensive recombination between subspecies (Crowley et al. 2024, Sivertsen et al. 2025).

Additionally to this challenging strain-level heterogeneity in *Fn*, the oral cavity, which acts as the bacterium’s endogenous niche, is equally as complex in terms of potential TME interactions and their combined pro-tumourigenic effect (Niño et al. 2022). For example, *Porphyromonas gingivalis* (*P. gingivalis*) represents a pathobiont that exists in a balanced immunoinflammatory state and whose pathogenicity is influenced by non- and modifiable factors, such as lifestyle and the host’s immune system (Gallimidi et al. 2015). Co-infection of a murine OSCC model with *P. gingivalis* and *Fn* produced a notable difference in the expression of IL-6 compared to either pathogen alone, resulting in enhanced tumour proliferation and invasiveness. Evidence supplied by spatial profiling in CRC tissues identified both *P. gingivalis* and *Fn* as being associated with the same cell clusters and significantly upregulating the signalling pathways involved in EMT, inflammation, and metastasis (Niño et al. 2022). This polymicrobial diversity within the TME and possible lack of understanding concerning the role of individual microbes complicates the study and replication of *Fn-*specific tumour interactions.

The increased understanding of the oncogenic roles of *Fn*-associated virulence factors carries substantial therapeutic potential (Table 3). However, the benefits of anti-*Fn* intervention are not currently clear due to the bacterium’s mutualistic role in the oropharynx, its potential for antimicrobial resistance (AMR), and the lack of reporting on *Fn*-targeting initiatives. As data from various regions illustrate the level of AMR for *Fn* to be 6.7%–25% for clindamycin and 7%–50% for fluoroquinolones, susceptibility testing and tightly regulated therapies are of the essence to mitigate the spread of resistance genes (Kowsarkhizi et al. 2025). Prevention tactics, such as *Fn*-directed vaccines, phage-based therapeutics, and microbial ecosystem replacement therapy, may be premature and even harmful until evaluated properly (Brennan and Garrett 2019). Similar to AMR, phage therapy optimization for genetically diverse *Fn* strains and proper integration into existing regimens will also likely call for an extensive amount of regulatory compliance. Even though the bacterium has been proposed to serve as a biomarker for the stratification of patients based on *Fn* burden, the concept remains unvalidated, likely due to high heterogeneity (Kowsarkhizi et al. 2025). Further research should incorporate the symbiotic impact of *Fn* on the host’s metabolism and immunity, accounting for the plausible reciprocal outcomes to the microbiome made by preventative and immunotherapies. These factors support the research need for a well-investigated, narrow-spectrum drug specific for oncogenic *Fn* strains and tumour tissues, making the intervention safe and biologically relevant.

**Table 3:** *Fn*-associated proteins and pathways, targetable therapeutically.

Target & tumour	Therapeutic targeting	References
CCR6-CCL20 axis in OSCC, mouse model	CCR6, expressed in T regulatory and helper 17 cells, is exclusively activated by the CCL20 chemokine. The use of the anti-CCL20 antibodies combined with radiotherapy reduced tumour volume by 67%.	Rutihinda et al. 2022
OMVs in OSCC, mouse model	OMVs are secreted for communication. The addition of an autophagy inhibitor, chloroquine, attenuated the vesicles’ activity of promoting cell migration and invasion (i.e. 35% fewer migrating cells).	Chen et al. 2024
FadA in CRC, mouse model	FadA is an adhesin which modulates E-Cadherin and $\beta $-catenin signalling, among others. Prevention of FadA binding with an angiogenesis inhibitor, genistein, significantly reduced FadA-associated inflammatory gene expression.	Guo et al. 2020
TIGIT checkpoint receptor in CRC, mouse model	TIGIT, a co-inhibitory or exhaustion receptor, is expressed in NK and T cells. TIGIT blockade with monoclonal antibodies resulted in a 75% smaller tumour volume, 50%+ longer survival and a 1–2-fold increase in the proliferating cell number.	Zhang et al. 2018

Note: These are only some examples of how the detection of *Fn*-abundant oral and colorectal tumours may direct treatment selection.

## Conclusion

This review aimed to describe the role of *Fn* in the TME and carcinogenesis—specifically, throughout malignancy development, escape and dissemination. The microbiome and its members play a role in both OCCs and CRCs, as evidenced by the activity and adaptation of *Fn* at different stages of OCC and CRC. The bacterium’s involvement in tumourigenesis can be linked to DNA damage accumulation, glucose uptake, and abnormal proliferation, driven by the changes in signalling and expression regulation. Virulence factors of *Fn* lead to chronic inflammation within the TME, similar to IBD and periodontitis, but also contribute towards an anti-tumour immunity to escape the host’s innate system. These *Fn*-associated changes in MDSC differentiation result in the suppression of T lymphocytes and, possibly, many more cascades of adaptive immunity. Lastly, *Fn* has been found at the metastatic sites, confirming its involvement in tissue remodelling and EMT mechanisms, altered by the *Fn*-directed agents and cellular response to infection. Given the decreasing price of genomic sequencing and advances in pharmacology, our understanding of the opportunistic *Fn* and the consequences of its symbiosis disruption will direct its proper targeting. However, before the gaps addressed in this review are investigated and resolved, therapeutic targeting of *Fn* in OCC and CRC should be approached with care.

## Supplementary Material

xtag002_Supplemental_File
