# Supplementary material for: The role of Fusobacterium nucleatum in the tumour microenvironment and carcinogenesis of oral and colonic malignancies
Source: FEMS Microbes. 2026 Jan 10;7:xtag002. doi: 10.1093/femsmc/xtag002 (PMC12853310; doi:10.1093/femsmc/xtag002)
Supplement: xtag002_Supplemental_File [file xtag002_supplemental_file.docx]

Review and synthesis of the role in malignancies, genomic analysis, and therapeutic potential of a gram-negative oral pathogen, Fusobacterium nucleatum.
